# Phase I/II Trial of Carboplatin, *Nab*-paclitaxel, and Pembrolizumab for Advanced Non–Small Cell Lung Cancer: Hoosier Cancer Research Network LUN13-175

**DOI:** 10.1093/oncolo/oyad180

**Published:** 2023-06-30

**Authors:** Ryan D Gentzler, Nisha A Mohindra, Shadia I Jalal, Karen L Reckamp, Richard D Hall, Nasser H Hanna, Young Kwang Chae, Marianna Koczywas, Irene B Helenowski, Jyoti D Patel

**Affiliations:** Department of Medicine, Division of Hematology/Oncology, University of Virginia Cancer Center, Charlottesville, VA, USA; Department of Medicine, Division of Hematology and Oncology, Robert H. Lurie Comprehensive Cancer Center, Northwestern University, Chicago, IL, USA; Department of Medicine, Division of Hematology/Oncology, Indiana University Melvin and Bren Simon Cancer Center, Indianapolis, IN, USA; Department of Medicine, Division of Medical Oncology, Cedars-Sinai Medical Center, Los Angeles, CA, USA; Department of Medicine, Division of Hematology/Oncology, University of Virginia Cancer Center, Charlottesville, VA, USA; Department of Medicine, Division of Hematology/Oncology, Indiana University Melvin and Bren Simon Cancer Center, Indianapolis, IN, USA; Department of Medicine, Division of Hematology and Oncology, Robert H. Lurie Comprehensive Cancer Center, Northwestern University, Chicago, IL, USA; Department of Medical Oncology & Therapeutics Research, City of Hope Comprehensive Cancer Center, Duarte, CA, USA; Department of Preventive Medicine, Northwestern University, Chicago, IL, USA; Department of Medicine, Division of Hematology and Oncology, Robert H. Lurie Comprehensive Cancer Center, Northwestern University, Chicago, IL, USA

**Keywords:** lung cancer, adenocarcinoma, carboplatin, *nab*-paclitaxel, pembrolizumab, immunotherapy

## Abstract

**Background:**

Combination chemotherapy and immunotherapy regimens have significantly improved survival for patients with previously untreated advanced non–small cell lung cancer (NSCLC). Improvements in overall survival (OS) in two separate pembrolizumab trials have demonstrated survival improvements over chemotherapy alone, regardless of PD-L1 status. The optimal chemotherapy backbone for combination with immunotherapy is unknown. We hypothesized *nab*-paclitaxel may be a well-suited platinum partner to use in combination with checkpoint inhibitor therapy for both adenocarcinoma and squamous histology and conducted a phase I/II trial to assess the efficacy of this regimen in advanced NSCLC.

**Methods:**

Adult patients with previously untreated, stage IIIB/IV NSCLC (any histology) with an Eastern Cooperative Oncology Group performance status of 0-1, any PD-L1 expression, and no *EGFR* mutations or *ALK* translocations, received carboplatin area under the curve (AUC) 6 day 1, *nab*-paclitaxel 100 mg/m^2^ days 1, 8, 15, and pembrolizumab 200 mg day 1 q21 days for 4 cycles followed by maintenance pembrolizumab q3w. Co-primary endpoints were progression-free survival (PFS) and overall response rate (ORR).

**Results:**

Forty-six evaluable patients enrolled, 14 in phase I and 32 in phase II, from June 2015 to July 2018 with a median duration of follow-up of 35.4 months. Median time from enrollment to data lock was 42 months. In the ITT population, the ORR was 35%, median PFS was 5.6 months (95% CI, 4.6-8.2), and median OS was 15.4 months (CI, 12.4-28.1). There were no statistical differences in PFS or OS by PD-L1 status. The 2- and 3-year landmark OS rates were 33% and 24%, respectively.

**Conclusion:**

Carboplatin, *nab*-paclitaxel, and pembrolizumab are a safe and effective regimen for patients with both squamous and nonsquamous NSCLC. Although this study did not meet the prespecified endpoints, the median and landmark OS results are consistent with durable benefit of this regimen as seen in phase III trials for first-line treatment of advanced NSCLC.

## Teaser

This article reports the primary and long-term outcomes of the Hoosier Cancer Research Network LUN13-175 study.

**Implications for Practice**

This phase I/II trial for patients with advanced non–small cell lung cancer (NSCLC) is the first trial to report a taxane-based regimen for use in all NSCLC histology subtypes in combination with pembrolizumab. Previous trials used a mix of either paclitaxel or *nab*-paclitaxel and restricted taxanes to patients with squamous histology. This study provides additional insights into the safety and tolerability of the *nab*-paclitaxel combination regimen used uniformly in all patients. Having these data in nonsquamous histology is important for clinical practice, particularly when the use of pemetrexed may be limited due to renal impairment. This study demonstrates long-term overall survival that compares favorably to historical controls and is comparable to long-term survival seen in contemporary phase III combination trials. Notably, this trial enrolled a higher proportion of African Americans, which is more representative of the general population than previously published in first-line immunotherapy trials for NSCLC.

## Introduction

Lung cancer is the leading cause of annual cancer-related mortality. Inhibitors of the programmed death 1 receptor (PD-1) and its ligand PD-L1 have demonstrated efficacy and improved survival for patients with non–small cell lung cancer (NSCLC). Pembrolizumab is an anti-PD-1 antibody that improved overall survival (OS) for metastatic NSCLC in the second-line setting after platinum-doublet chemotherapy^1^ and for treatment-naive tumors with PD-L1 expression of ≥50% or ≥1%.^2,3^ Pembrolizumab has also demonstrated OS improvements in combination with platinum-doublet chemotherapy, regardless of PD-L1 status.^4,5^ The optimal chemotherapy backbone for combination with immunotherapy is unknown. Chemotherapy may augment the immune response through increased antigen presentation and reduction of myeloid-derived suppressor and T-regulatory cells, which are negative regulators of the immune system.^6,7^ We sought to evaluate the safety and efficacy of carboplatin, *nab*-paclitaxel, and pembrolizumab for patients with treatment-naïve, advanced, NSCLC, based on data that showed carboplatin and *nab*-paclitaxel had higher response rates and similar progression-free survival (PFS) to carboplatin and paclitaxel^8^ but does not require corticosteroids, which may impair an immune response through downregulation of T-cell activation^9^ and expansion of T-regulatory cells.^10^ We hypothesized *nab*-paclitaxel may be a well-suited platinum partner to use in combination with checkpoint inhibitor therapy for both adenocarcinoma and squamous histology. This regimen has subsequently been used in KEYNOTE 407,^5,11^ a phase III randomized trial for patients with squamous cell NSCLC, and in IMpower130^12^ and IMpower131^13^ with atezolizumab for nonsquamous and squamous NSCLC, respectively. This regimen demonstrated OS improvement compared to chemotherapy alone in both the KEYNOTE 407 and IMpower130 trials. Here we report the primary and long-term outcomes of this phase I/II study, the Hoosier Cancer Research Network (HCRN) LUN13-175.

## Methods

### Patients

Patients who were at least 18 years of age with pathologically confirmed stage IIIB/IV (AJCC 7th ed.) NSCLC, not amenable to definitive local therapy, were eligible for enrollment. Patients were Eastern Cooperative Oncology Group performance status 0-1 and had received no prior treatment for metastatic disease. Patients with known *EGFR* mutations or *ALK* gene rearrangements, prior malignancy in preceding 12 months, HIV, hepatitis B/C, liver cirrhosis, history of pneumonitis, active autoimmune disorder requiring immunosuppressive treatment in the preceding 2 years, radiation treatment within 2 weeks prior to enrollment, and pre-existing grade 2 or higher peripheral neuropathy were excluded. Patients without genomic testing results were permitted to enroll. Patients with treated brain metastases were allowed to enroll provided they had clinical stability for 4 weeks after treatment and were off steroids for at least 2 weeks.

The study protocol was approved by local institutional review boards at each institution and the trial was conducted in accordance with the Declaration of Helsinki and Good Clinical Practice standards. Patients provided written informed consent prior to enrollment and participation.

### Trial Design and Treatment

HCRN LUN13-175 was a multi-institutional phase I/II investigator-initiated trial evaluating the combination of carboplatin, *nab*-paclitaxel, and pembrolizumab in patients with treatment-naïve stage IIIB/IV NSCLC. The phase I primary objectives were to determine the recommended phase II dose schedule (RP2D) and evaluate the safety and tolerability of this combination therapy. For phase II, the co-primary objectives were to evaluate PFS and objective response rate (ORR). Secondary objectives were to describe OS, antitumor activity, safety and tolerability, and PD-L1 as a predictor of PFS.

The phase I portion of the trial planned to enroll 12 patients in a safety lead-in cohort. If 4 or more patients experienced dose-limiting toxicity (DLT), a second cohort of 12 was planned. In this cohort, pembrolizumab would be delayed until cycle 2 of treatment. If 3 or fewer patients experienced DLT in cohort 1 (or cohort 2, if necessary), the trial would proceed with a single-arm phase II cohort of 43 patients at the RP2D. Dose-limiting toxicities were defined as a missed dose of pembrolizumab, persistent grade 2 uveitis or pneumonitis, any symptomatic grade 3 adverse event (AE) lasting 3 days despite optimal therapy, or any grade 4 AE attributed to pembrolizumab or the combination of pembrolizumab and chemotherapy. If a second cohort was necessary in phase I, all treatment would be the same with the exception that pembrolizumab would start with cycle 2 of treatment, instead of cycle 1, to reduce the number of cycles of overlap with chemotherapy.

For phase II, cohort 1, patients would be treated with pembrolizumab 2 mg/ kg on day 1, *nab*-paclitaxel 100 mg/m^2^ days 1, 8, and 15, and carboplatin area under the curve (AUC) 6 on day 1 every 3 weeks for a total of 4 cycles. Patients achieving stable disease or a partial response after 4 cycles would then proceed with pembrolizumab maintenance at 2 mg/kg every 21 days for up to 2 years (total of 34 cycles). For phase II, pembrolizumab was to be dosed based on the RP2D from phase I; however, due to changes in FDA approval of pembrolizumab during the course of the trial, flat dosing of 200 mg in lieu of the 2 mg/kg weight-based dosing was used for all patients in the phase II portion of the trial. Treatment for all patients continued until disease progression, unacceptable toxicity, investigator decision, patient withdrawal of consent, or completion of 2 years of treatment.

### Assessments

Archival or fresh tumor biopsies were obtained prior to cycle 1 day 1 of treatment for all patients. A second biopsy was obtained at the end of cycle 4 or at the time of progression, whichever occurred first. Tissue at both time points was assessed for PD-L1 expression by IHC using the 22C3 (Dako) antibody. Expression was categorized according to tumor proportion score (TPS; the percentage of tumor cells with membranous PD-L1 staining). Treating physicians could opt out of post-treatment biopsies if it was felt unsafe per physician discretion or if significant tumor response would result in low yield with minimally invasive techniques (eg, computed tomography [CT] or ultrasound-guided core needle biopsy). Later, in the course of the study when local PD-L1 testing became standard of care, a protocol amendment allowed for local PD-L1 testing using the 22C3 antibody to be used for study entry without tissue submission for central PD-L1 testing. Tumor imaging was performed every 6 weeks for the first 12 weeks, and then every 9 weeks thereafter. Response was assessed according to Response Evaluation Criteria in Solid Tumors (RECIST) version 1.1. Adverse events and laboratory abnormalities were graded according to the National Cancer Institute Common Terminology Criteria for Adverse Events version 4.0. Patients were contacted every 12 weeks for survival follow-up.

### Endpoints

For phase I, the primary endpoints were RP2D, safety, and tolerability. Progress-free survival and ORR were co-primary endpoints for phase II. Secondary endpoints were PFS (phase I only), OS, PFS by PD-L1 expression, and safety/tolerability in the overall trial population.

### Statistical Analyses

For the phase I portion of the study, the RP2D was defined as the dosing schedule of pembrolizumab resulting in less than 33% DLT rate assessed after the completion of 2 cycles. The RP2D was defined as the dosing in the cohort with <4 subjects with DLTs.

For the phase II portion of the study, co-primary endpoints were PFS and overall response rate (ORR). Recruitment of 43 patients over a 12-month period would be required to demonstrate the hypothesized improvement in median PFS to 9.0 months from a historical control of 6.0 months, with a 1-sided α of 0.05 and 80% power, assuming an exponential distribution of the PFS. Kaplan–Meier methodology was used to summarize the PFS, and a median PFS would be reported with a 95% confidence interval. The trial outcome for PFS would be considered successful if the 95% 1-sided confidence limit for the median is greater than 6 months. For response rate, a single-stage, single-arm design assumed a historical control response rate of 31% and hypothesized response rate of 50% with the same type I and type II error rates. The success of the study would be defined as a response being achieved in at least 19 of 43 patients. The analysis of both co-primary outcomes used an intention-to-treat approach from the time of registration. After 32 patients were enrolled in phase II, the trial closed due to slow accrual after FDA approval of similar chemotherapy and pembrolizumab regimens. Analyses for PFS were performed for phase II alone as well as phase I and II combined.

Exploratory biomarker analyses were planned on archival tissue and on-study treatment biopsies. PD-L1 expression would be evaluated using TPS and categorized as positive if 1% or higher expression was identified. The distribution of PFS using Kaplan–Meier method would be summarized based on PD-L1 expression and compared using 1-sided log-rank test. Nominal *P*-values will be reported for this comparison.

## Results

### Patients and Treatments

**Figure 1.**
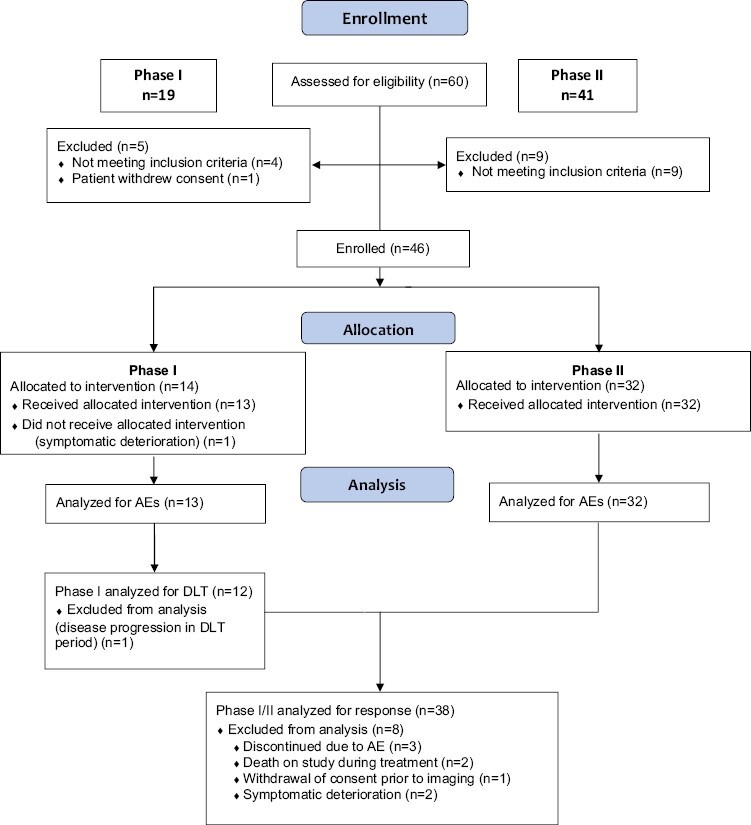
CONSORT flow diagram. CONSORT diagram for all patients assessed for eligibility for the trial. Phase I is depicted on the left side of diagram and phase II on the right. AEs, adverse events; DLT, dose-limiting toxicities.

Between June 2015 and July 2018, 46 patients were enrolled, 14 to phase I and 32 to phase II, at 4 sites in the United States (Fig. 1). At the time of data lock, the median duration of follow-up was 35.4 months, including censored patients. Median time from enrollment to data lock for all patients was 42 months. The baseline characteristics of phase I and II portions of the trial are shown in Table 1. PD-L1 expression (TPS) by <1%, 1%-49%, and ≥ 50% cutoffs were 43%, 34%, and 23%, respectively.

**Table 1:** Patient characteristics

	Overall*,* n (%)	Phase I*,* n (%)	Phase II*,* n (%)
N	46	14	32
Age (years)^a^	65.5 (39,79)	66 (45,76)	65.5 (39,79)
Gender			
Female	22 (48%)	9 (64%)	13 (41%)
Male	24 (52%)	5 (36%)	19 (59%)
Race			
Asian	1 (2%)	0 (0%)	1 (3%)
Black or African American	6 (13%)	1 (7%)	5 (16%)
White	35 (76%)	9 (64%)	26 (81%)
Unknown	4 (9%)	4 (29%)	0 (0%)
Ethnicity			
Hispanic or Latino	0 (0%)	0 (0%)	0 (0%)
Non-Hispanic	42 (91%)	11 (79%)	31 (97%)
Unknown	4 (9%)	3 (21%)	1 (3%)
Histology			
Adenocarcinoma	21 (46%)	10 (71%)	11 (34%)
Squamous cell carcinoma	21 (46%)	4 (29%)	17 (53%)
Non*–*small cell carcinoma, NOS	1 (2%)	0 (0%)	1 (3%)
Poorly differentiated carcinoma	3 (7%)	0 (0%)	3 (9%)
Smoking, cigarettes per day^a^	20 (0.03, 60)	20 (0.05, 40)	20 (0.03, 60)
ECOG performance status			
0	14 (31%)	6 (43%)	8 (26%)
1	31 (69%)	8 (57%)	23 (74%)
PD-L1, baseline			
<1%	19 (43%)	4 (31%)	15 (48%)
1-49%	15 (34%)	4 (31%)	11 (35%)
≥50%	10 (23%)	5 (38%)	5 (16%)
Not evaluable	2	1	1
Nab-paclitaxel, cumulative dose (max = 1200 mg m^−2^)			
<800 mg m^*−*2^	7 (16%)	1 (8%)	6 (19%)
≥800 mg m^*−*2^	38 (84%)	12 (92%)	26 (81%)

Baseline characteristics and cumulative dose of *nab*-paclitaxel received.

^a^Median (minimum, maximum).

Abbreviations: ECOG, Eastern Cooperative Oncology Group; NOS, not otherwise specified.

### Phase I Dose Confirmation

In the phase I portion of the study, 14 patients were enrolled. One withdrew due to symptomatic deterioration prior to study treatment, and one withdrew prior to the end of the DLT period due to disease progression and was replaced. In total, 13 patients were evaluable for AE reporting and 12 evaluable for DLTs. There were 2 DLTs, 1 grade 4 hyperglycemia, 1 grade 3 neutropenia. All AEs and DLTs were reviewed by the data safety review committee and the study proceeded with phase II at the RP2D of pembrolizumab 2 mg/kg starting cycle 1 concurrently with chemotherapy. Prior to initiating phase II, the study was amended to use the FDA-approved pembrolizumab 200 mg flat dose in lieu of the 2 mg/kg dose.

### Safety

Treatment-related AEs were reported in 82% of all patients enrolled (Table 2). Fatigue, neutrophil decrease, platelet decrease, anemia, and nausea were the most commonly reported AEs with at least possible attribution to study drugs. The most common grade 3 or higher AEs occurring in more than 1 patient were neutrophil count decrease (73%), anemia (31%), and platelet count decrease (27%). Two patients died while on treatment, 1 from unspecified causes, and 1 from cardiac arrest. In both cases, AEs were deemed to be unlikely or unrelated to study treatments.

**Table 2.** Adverse Events

	All, n (%)	Grade 1/2, n (%)	Grade 3/4, n (%)
Fatigue	37 (82)	33 (73)	4 (9)
Neutrophil decrease	37 (82)	4 (9)	33 (73)
Platelet decrease	31 (69)	19 (42)	12 (27)
Anemia	27 (60)	13 (29)	14 (31)
Nausea	25 (56)	24 (53)	1 (2)
Alopecia	20 (44)	20 (44)	
Anorexia	17 (38)	15 (33)	2 (4)
Diarrhea	17 (38)	16 (36)	1 (2)
WBC decrease	13 (29)	5 (11)	8 (18)
Rash	12 (27)	11 (24)	1 (2)
Dysgeusia	10 (22)	10 (22)	0 (0)
Vomiting	10 (22)	10 (22)	0 (0)
Constipation	9 (20)	9 (20)	0 (0)
Hypokalemia	9 (20)	8 (18)	1 (2)
Peripheral sensory neuropathy	9 (20)	9 (20)	0 (0)
Arthralgia	6 (13)	5 (11)	1 (2)
Administration site conditions, other	6 (13)	5 (11)	1 (2)
Dizziness	5 (11)	5 (11)	0 (0)
Fever	5 (11)	5 (11)	0 (0)
Hyponatremia	5 (11)	4 (9)	1 (2)
Hypothyroidism	5 (11)	5 (11)	0 (0)
Lymphocyte count decreased	5 (11)	3 (7)	2 (4)
Mucositis oral	5 (11)	5 (11)	0 (0)

Treatment-related adverse events (AEs) occurring in at least 10% of patients.

Abbreviations: WBC, white blood count.

### Outcomes

**Figure 2.**
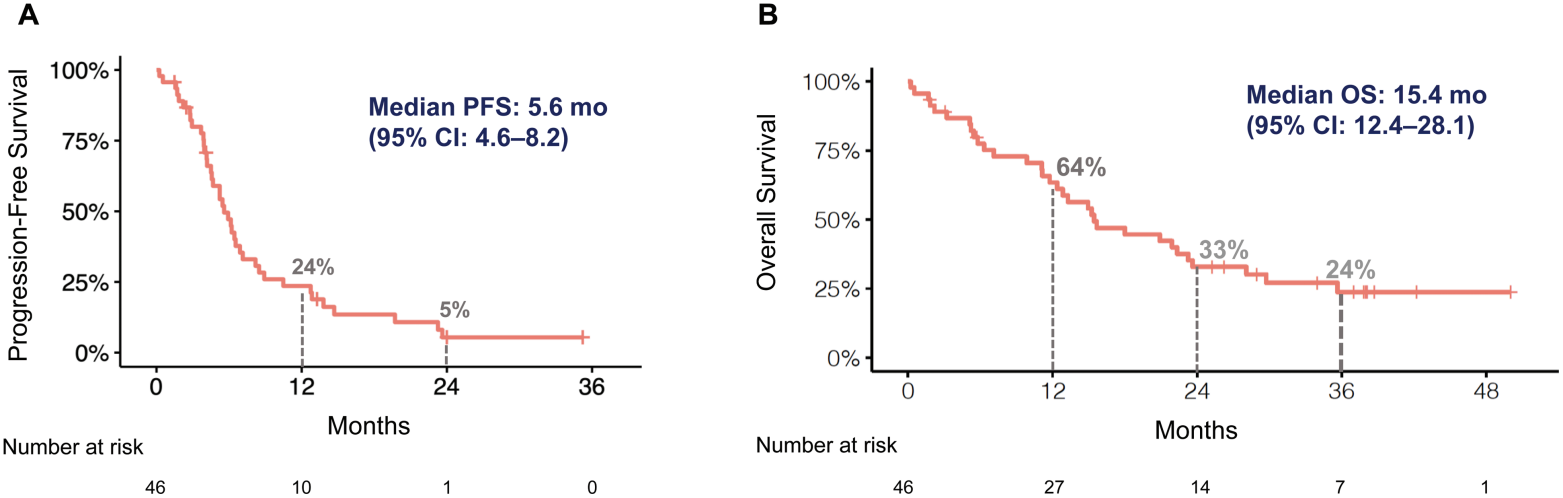
Progression-free survival and overall survival, all patients. (**A**):. Progression-free survival. (**B**):Overall survival. PFS, progression-free survival; OS, overall survival; CI, confidence interval.

**Figure 3.**
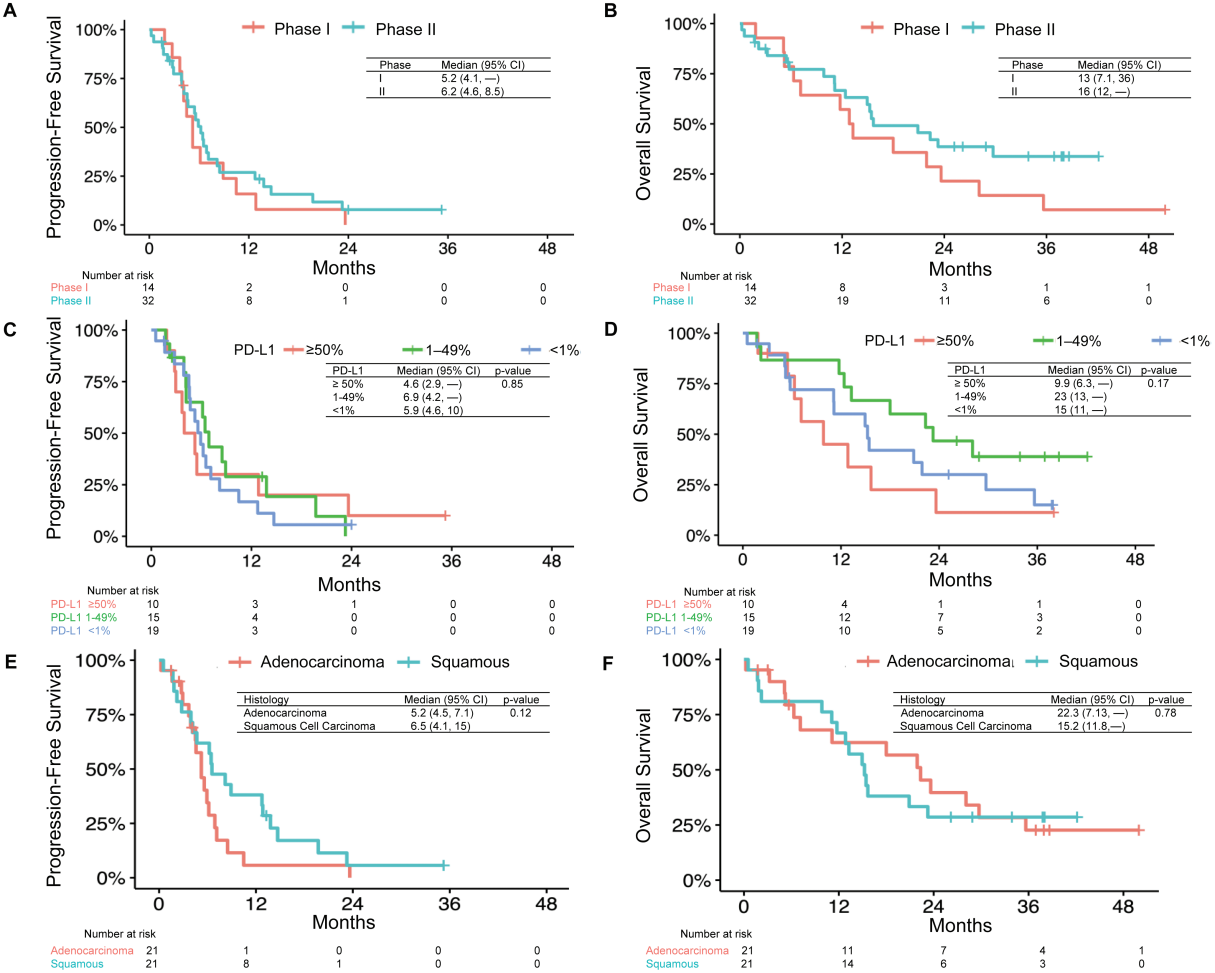
Progression-free survival and overall survival outcomes by subgroups. PFS by (**A**) phase of trial, (**C**) PD-L1 tumor proportion score, and (**E**) histology. OS by (**B**) phase of trial, (**D**) PD-L1 tumor proportion score, and (**F**) histology. PFS, progression-free survival; OS, overall survival; CI, confidence interval.

The median PFS for all patients was 5.6 months (95% CI, 4.6-8.2 months, Fig. 2A). At 12 months and 24 months, PFS was 23.6% (95% CI 13.7%-40.5%) and 5.4% (95% CI, 1.4%-20.3%) respectively. PFS was similar for patients enrolled in phase I (median 5.2 months, 95% CI, 4.1-NR) and phase II (median 6.2 months, 95% CI 4.6-8.5 months, *P* = .48) cohorts (Fig.3A). There were no significant differences in PFS between 3 PD-L1 subgroups (*P* = .85, Fig. 3C) or histology (*P* = .12, Fig. 3E). Patients with NSCLC not otherwise specified (NOS) histology had numerically lower PFS of 4.8 months than those with adenocarcinoma (5.2 months) or squamous cell carcinoma (6.5 months).

Overall survival was 15.4 months (95% CI, 12.4-28.1 months, Fig. 2B). Landmark survival rates at 12, 24, and 36 months were 63.5%, 32.9%, and 23.8%, respectively. There was no significant difference (*P* = .15) in OS for patients enrolled in phase I, median OS of 13 months, and those in phase II with a median of 16 months (Fig. 3B). There were no differences in OS based on PD-L1 status (*P* = .17, Fig. 3D) or histology (*P* = .78, Fig. 3F).

**Figure 4.**
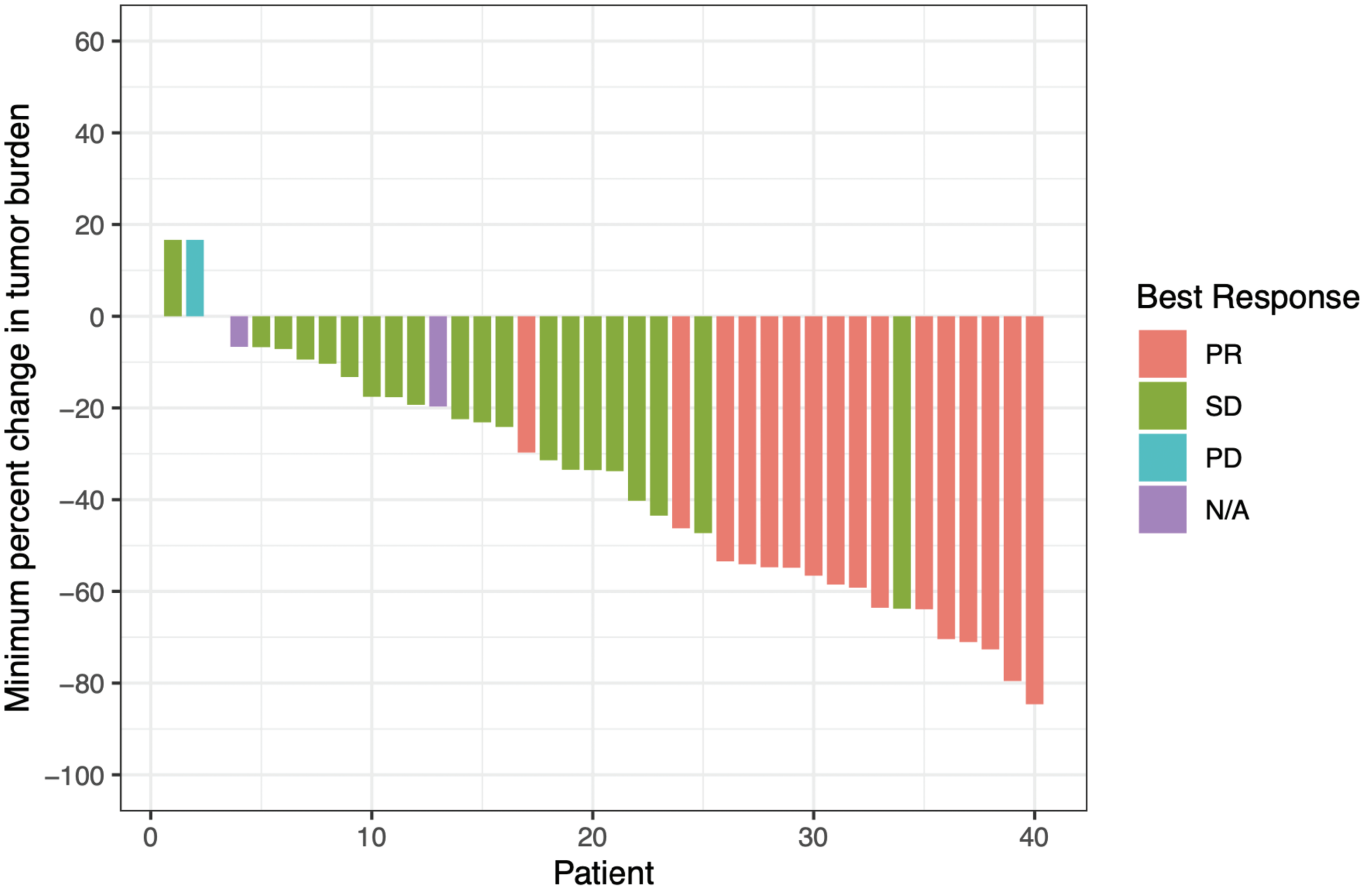
Waterfall plot of best change in tumor dimensions. Waterfall plot of best change in tumor dimensions including all trial patients with baseline and at least 1 on-study CT assessment. Bars represent a single patient and are color coded by best response by Response Evaluation Criteria in Solid Tumors 1.1 criteria. PR, partial response; SD, stable disease; PD, progression of disease; N/A, not available.

Sixteen of 46 patients achieved a partial response, corresponding to an ORR of 34.8%. Twenty-one patients (45.7%) had stable disease, 1 (2.2%) had progression of disease, and 8 (17.4%) were unevaluable for response. Of the 40 patients with at least 2 imaging assessments, 37 (93%) had a reduction in target lesion measurements (Fig. 4). The ORR for PD-L1 subgroups was 31.6% for PDL1 <1%, 53.3% for PDL1 1%-49%, and 20.0% for PDL1 ≥50%.

### Exploratory Analyses

**Figure 5.**
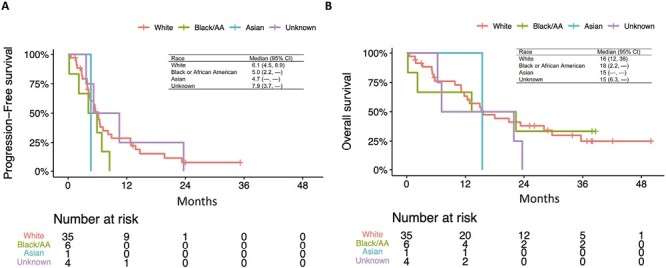
Progression-free survival (PFS) and overall survival (OS) outcomes by race. (**A**): PFS subgroups by race. (**B**): OS subgroups by race. AA, African American; CI, confidence interval.

Unplanned, post-hoc exploratory analyses were conducted to evaluate outcomes by race, cumulative *nab*-paclitaxel dose, and changes in PD-L1 for paired biopsies. Median PFS was 6.1 months for patients of White race, 5.0 months for Black/African American race, 4.7 months for Asian race, and 7.9 for those with unknown race (Fig. 5). Median OS was numerically highest among the Black/African American subgroup at 18 months. Subgroups by race were small, but there were no clear significant differences in PFS (*P* = .45) or OS (*P* = .79) between patients of different races. Of the African Americans enrolled, 3 of 6 had PR (50% response rate), 1 had SD and 2 were unevaluable for response.

Given notable hematologic toxicity requiring dose reductions, an exploratory analysis of outcomes based on cumulative *nab*-paclitaxel dose was performed. The maximum possible dose was 1200 mg/m^2^ over the 4 cycles (100 mg/m^2^ on days 1, 8, and 15 of each of the 4 cycles). Three patients had received less than 400 mg/m^2^ and 4 patients received between 400 and 799 mg/m^2^, representing less than 2 of the 3 prescribed doses at 100 mg/m^2^ per cycle. Those who received at least 800 mg/m^2^ of *nab*-paclitaxel had improved PFS, median 6.2 months versus 1.8 months for those receiving <800 mg/m^2^ (*P* < .001, Figure S1). OS was also improved for those receiving at least 800 mg/m^2^, achieving a median OS of 21.0 months vs. 1.8 months for those receiving <800 mg/m^2^ (*P* = .017). There was also a significant difference in OS when comparing 3 subgroups receiving >800 versus 400-799 versus <400 mg/m^2^ (*P* < .001). For 4 patients who received 400-799 mg/m^2^ of *nab*-paclitaxel, the median OS was 19 months.

### Correlative Studies

**Figure 6.**
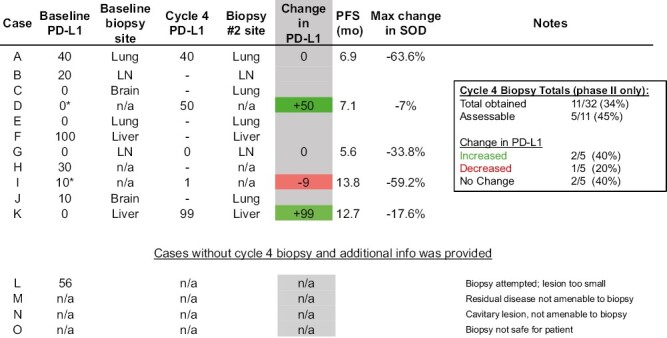
Comparison of pretreatment baseline PD-L1 and cycle 4 PD-L1. PD-L1 tumor proportion scores (TPS) as assessed by central lab with Dako 22C3 from tumor biopsies. Cases with “-” had tissue submitted that could not be assessed for PD-L1. n/a indicates tissue was not available. Baseline cases with “*” indicate that tissue was not available for central PD-L1 testing and PD-L1 testing results were per local laboratory. PFS, progression-free survival; SOD, sum of diameters; mo, months; LN, lymph node.

Tumor biopsies were obtained during cycle 4 on the trial prior to the beginning of cycle 5. Biopsies were assessed for PD-L1 TPS. A total of 11 samples were collected and 5 were adequate for PD-L1 assessment (Fig. 6). Three of the 5 (60%) on-treatment biopsies had different PD-L1 expression compared to baseline pretreatment results. Two (40%) had higher PD-L1 than baseline, 1 (20%) had decreased PD-L1, and 2 (40%) were unchanged.

## Discussion

The results from our study demonstrate that carboplatin, *nab*-paclitaxel, and pembrolizumab are a safe regimen. Although there were 2 DLTs in the phase I portion, a grade 4 hyperglycemia type I diabetes event^14^ and a febrile neutropenia event, the regimen was safe and manageable. Hematologic toxicity was higher than expected and more common than in previously reported studies. Complete blood counts (CBCs) and AEs were collected weekly and may have artificially inflated these numbers relative to regimens with laboratory and AE assessments obtained at 3-week intervals. In the KEYNOTE 407 trial, weekly CBCs were only obtained in 40% of patients receiving *nab*-paclitaxel. Our protocol initially utilized specific criteria for dose reductions and delays per *nab*-paclitaxel dosing recommendations according to the FDA prescribing information, notably ANC <500 and platelets <50 for delays or dose reductions for days 8 and 15. These recommendations may have resulted in more frequent and higher-grade hematologic toxicity than seen in clinical practice where clinicians may reduce or hold doses for less-severe cytopenias. Later, amendments to the protocol allowed for dose adjustments and skipped doses of day 8 and day 15 *nab*-paclitaxel to be permissible per treating physician discretion, which was more feasible and in line with typical clinical practice. Our analysis of *nab*-paclitaxel dose received demonstrated improved outcomes with those receiving at least 800 mg/m^2^ over the course of cycles 1-4, the equivalent of at least 2 doses at 100 mg/m^2^ per cycle. Further research is needed to know whether there is further improvement in outcomes when patients receive >800 mg/m^2^ over 4 cycles.

The response rate of 30% and median PFS of 5.6 months did not meet prespecified targets for the success of the trial. However, the PFS and OS outcomes from this trial are similar to previously reported trials. When comparing survival outcomes by histology, the median OS of 22.3 months for the adenocarcinoma subgroup is comparable to results from the KEYNOTE 189 trial where median OS was 22.0 months and IMpower130 with median OS of 18.5 months. For squamous histology, median OS of 15.2 months is also similar to OS results from IMpower131 and KEYNOTE 407, which demonstrated median OS of 14.2 and 17.1 months, respectively. One key difference between this trial and the randomized phase III chemotherapy and immunotherapy combination trials was biomarker analysis required for enrollment. Although our trial required tissue for central PD-L1 testing, enrollment was not contingent on PD-L1 TPS results. Therefore, patients could immediately enroll and start treatment on our trial while the tissue was processed and shipped, unlike other studies that would have required tissue submission, central analysis, and eligibility confirmation prior to enrollment. It would have been more feasible for patients with symptomatic disease to enroll in this study compared to other trials where delays in treatment initiation would have been required to confirm eligibility. Since next-generation sequencing for genomic alternations was not required for enrollment, patients with mutations less likely to respond to immunotherapy may have been included in the study. Additionally, this trial enrolled NSCLC, regardless of histology. The trial enrolled 9% with poorly differentiated carcinoma or NSCLC NOS, and this group had a shorter median PFS of only 4.8 months, suggestive of a more aggressive disease state in this subgroup of patients.

Multiple prior studies have associated higher PD-L1 TPS with improved outcomes with pembrolizumab treatment. There was no association of PD-L1 TPS and PFS or OS in our study, although a small sample size may limit the ability to detect a difference. The distribution of PD-L1 expression in our study was skewed toward the PD-L1-negative with 43% of patient tumors having PD-L1 <1%, a higher proportion than those enrolled in KEYNOTE 407 and IMpower130. Our study attempted to evaluate the dynamic changes in PD-L1 after 4 cycles of chemotherapy and pembrolizumab. Despite being a protocol requirement when feasible, only 11 of 32 phase II samples were obtained and 5 were adequate for analysis. There was no central pathology assessment, but at least 1 patient is known to have no active disease in the biopsy specimen on local pathology assessment. Of the 5 assessed on-treatment samples, only 3 had baseline central PD-L1 results for comparison. The results in this limited sample provide 2 examples of a marked increase in PD-L1 expression. This may be a result of tumor heterogeneity, biopsy of different lesions, or demonstration of changes in PD-L1 expression throughout the course of treatment. Obtaining on-treatment biopsies remains a significant challenge for stage IV lung cancer trials.

This single-arm phase I/II trial was initially designed in 2013 before any clinical data were available on the safety or efficacy of immunotherapy and chemotherapy combinations. Therefore, a phase I trial to assess safety was planned prior to expanding to a single-arm phase II study. We hypothesized that *nab*-paclitaxel was an ideal platinum partner to study in combination with anti-PD-1 therapy due to the lack of need for corticosteroid premedication that is needed when administering other agents, such as pemetrexed or paclitaxel. Steroid-sparing antiemetic regimens can be used with *nab*-paclitaxel, and corticosteroid premedications were avoided in this protocol. During the conduct of this trial between June 2015 and July 2018, numerous phase II and phase III trials with various chemotherapy and checkpoint inhibitor immunotherapy combinations were completed and reported results, confirming the significant clinical activity of the strategy evaluated in this trial. Several of these trials used carboplatin and *nab*-paclitaxel as the chemotherapy backbone to which immunotherapy was added. The IMpower130 trial evaluated carboplatin, *nab*-paclitaxel, and atezolizumab in nonsquamous NSCLC and both IMpower131 and KEYNOTE 407 evaluated this same chemotherapy with atezolizumab and pembrolizumab, respectively, in squamous NSCLC. This trial remains the only study to evaluate a *nab*-paclitaxel and pembrolizumab combination in patients with nonsquamous NSCLC. This is an important distinction as pemetrexed may not be the ideal platinum partner for nonsquamous histology in clinical practice for cases with impaired renal function or prior exposure to pemetrexed for treatment of earlier-stage disease.

The use of a steroid-sparing chemotherapy regimen combined with immunotherapy does not appear to have resulted in higher response rates, PFS, or OS relative to other trials using chemotherapy that requires dexamethasone premedication. Chronic doses of prednisone 20 mg/day or higher have been associated with worse outcomes with immunotherapy in lung cancer,^15^ but transient exposure over a few days with other chemotherapy regimens may not be detrimental in the setting of steady drug exposure to pembrolizumab due to its half-life of 22 days.

Accrual was slower than planned for several reasons. First, accrual was halted for several months after the 12^th^ evaluable patient was enrolled in the first cohort of the phase I portion to evaluate safety in this cohort. Amendments for flat dosing of pembrolizumab were put in place before opening for the phase II portion. In the meantime, data from the randomized phase II KEYNOTE 021, cohort G trial^16^ were reported in 2016 and FDA approved in early 2017 for patients with nonsquamous histology. This approval and the subsequent KEYNOTE 189 phase III results may have contributed to slow enrollment as a similar regimen with pembrolizumab and chemotherapy was available off trial. Results from the KEYNOTE 407 trial for patients with squamous cell carcinoma were presented in mid-2018 that demonstrated an OS improvement with the addition of pembrolizumab to carboplatin and *nab*-paclitaxel or paclitaxel chemotherapy. Based on the positive results of this study, our study was closed to enrollment. The trial planned to enroll 43 patients in the phase II trial to evaluate efficacy at the RP2D, but despite closing early there were a combined 46 enrolled when including phase I and II cohorts treated with the same schedule. The key difference in the dose of pembrolizumab 2mg/kg during phase I and 200 mg flat dose in phase II is unlikely to result in different clinical outcomes between these 2 cohorts.

Our trial enrolled 13% African Americans, representing a higher proportion of racial minorities than represented in a pooled analysis of phase III first-line immunotherapy trials submitted to the FDA where only 1% of patients enrolled were Black.^17^ Although a small absolute number of patients, outcomes in African American patients appeared similar to outcomes of the overall trial population. Increased efforts to enroll patient populations representative of real-world demographics are needed to understand the true effectiveness of these therapies in different patient populations. The proportion of minority patients accrued in this trial demonstrates that it is feasible to increase minority accrual to clinical trials.

## Conclusions

Carboplatin, *nab*-paclitaxel, and pembrolizumab is a safe treatment regimen for patients with squamous and nonsquamous advanced NSCLC. Although the primary endpoints of ORR and PFS did not reach prespecified determinants of success, this regimen demonstrated long-term OS that compares favorably to historical controls and is comparable to long-term survival seen in contemporary phase III chemotherapy and immunotherapy combination trials.

## Supplementary Material

oyad180_suppl_Supplementary_Figure_S1

oyad180_suppl_Supplementary_Figure_S2

## Data Availability

The data underlying this article will be shared on reaso*nab*le request to the corresponding author.
