# Supplementary figures and images for: Phase I/II Trial of Carboplatin, Nab-paclitaxel, and Pembrolizumab for Advanced Non–Small Cell Lung Cancer: Hoosier Cancer Research Network LUN13-175
Source: Oncologist. 2023 Jun 30;29(1):47–56. doi: 10.1093/oncolo/oyad180 (PMC10769801; doi:10.1093/oncolo/oyad180)

## Slide 1
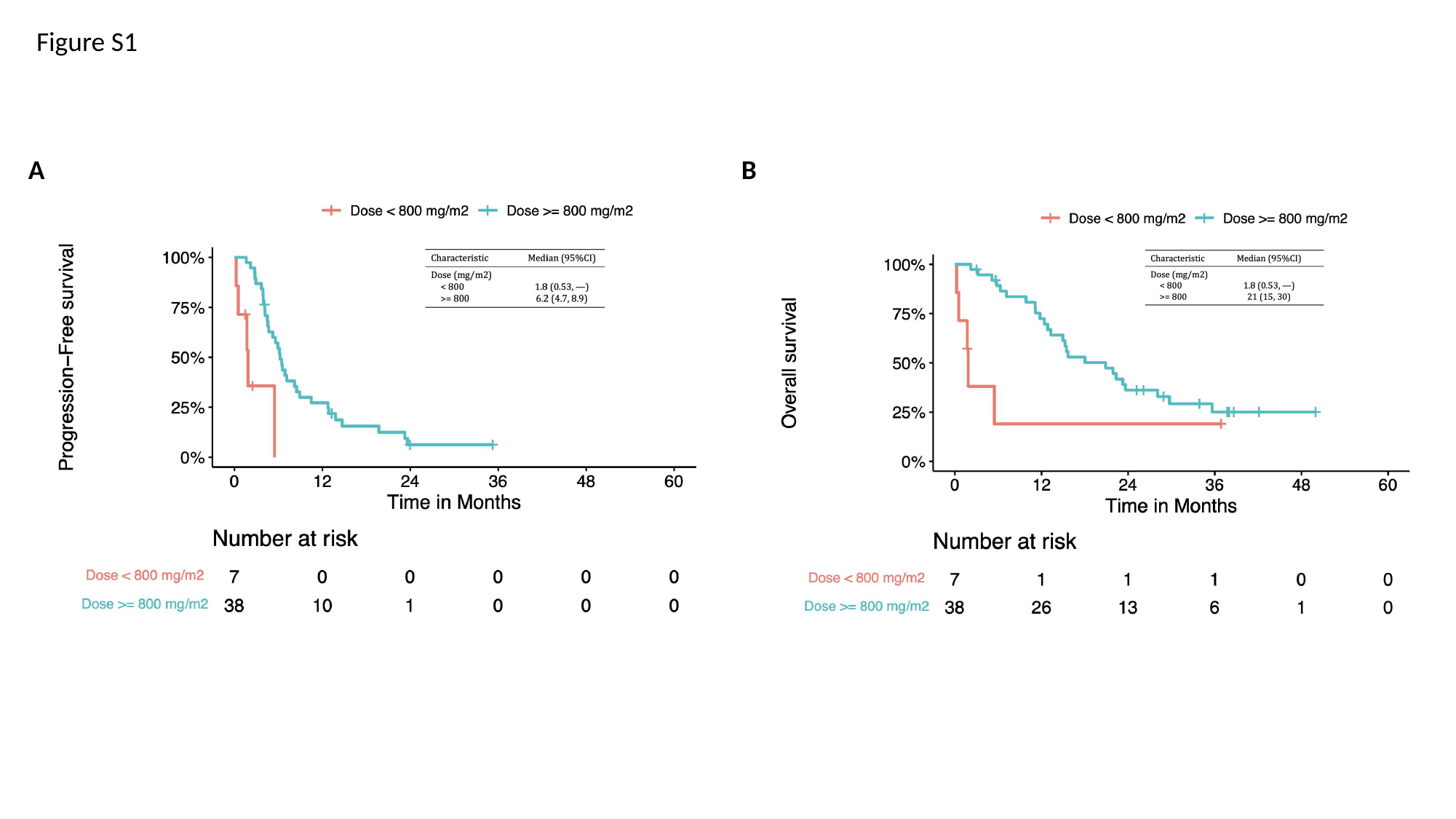

Figure S1
A
B

Supplement: oyad180_suppl_Supplementary_Figure_S1 [file oyad180_suppl_supplementary_figure_s1.pptx]
