# Supplementary material for: Phase I/II Trial of Carboplatin, Nab-paclitaxel, and Pembrolizumab for Advanced Non–Small Cell Lung Cancer: Hoosier Cancer Research Network LUN13-175
Source: Oncologist. 2023 Jun 30;29(1):47–56. doi: 10.1093/oncolo/oyad180 (PMC10769801; doi:10.1093/oncolo/oyad180)
Supplement: oyad180_suppl_Supplementary_Figure_S2 [file oyad180_suppl_supplementary_figure_s2.pptx]

## Slide 1
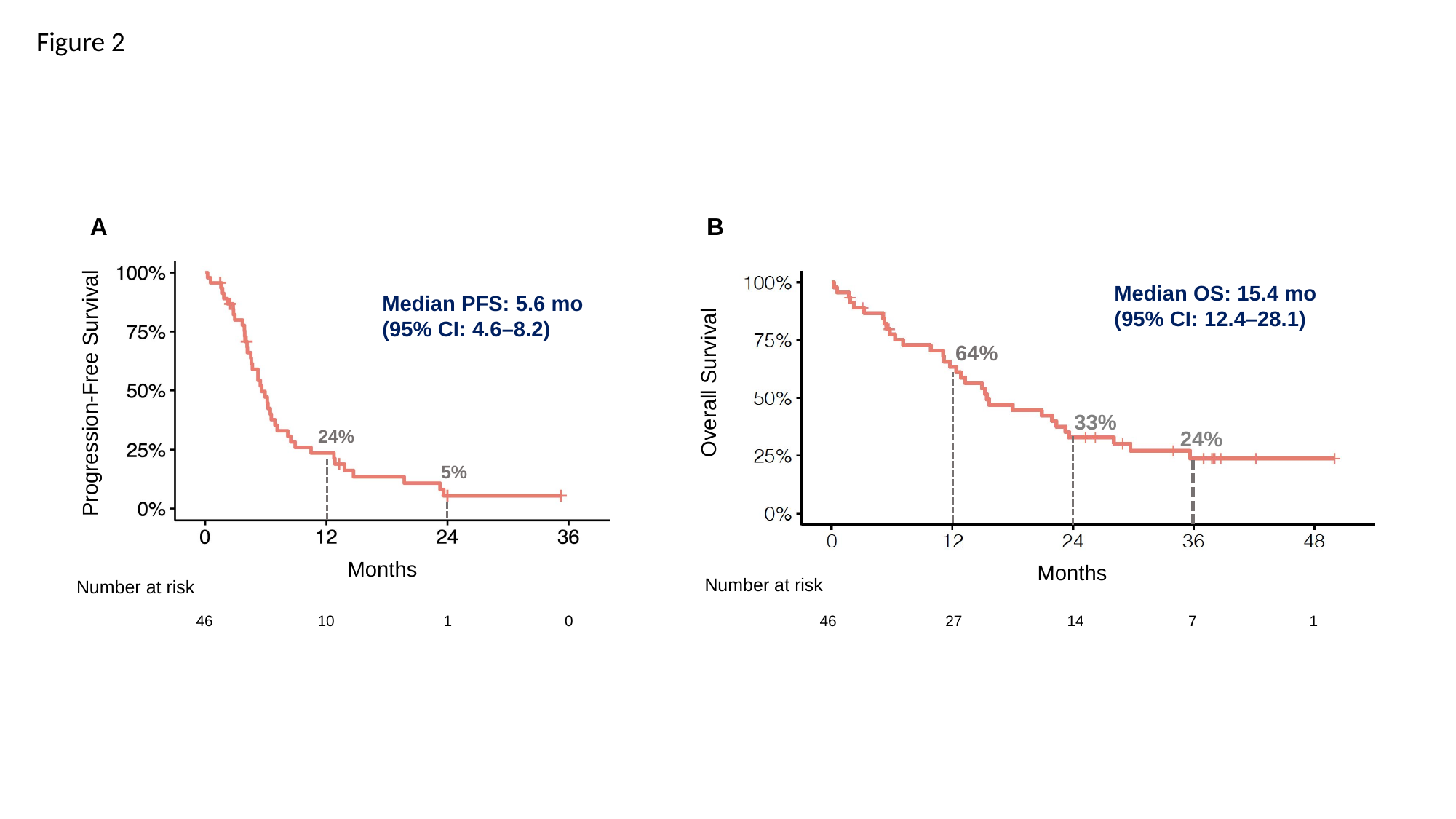

Figure 2
A
B
Progression-Free Survival
Median PFS: 5.6 mo
(95% CI: 4.6–8.2)
24%
5%
Months
Number at risk
Overall Survival
Median OS: 15.4 mo
(95% CI: 12.4–28.1)
64%
33%
24%
Months
Number at risk
 46	 27	 14 7 1
 46	 10	 1 0

## Slide 2
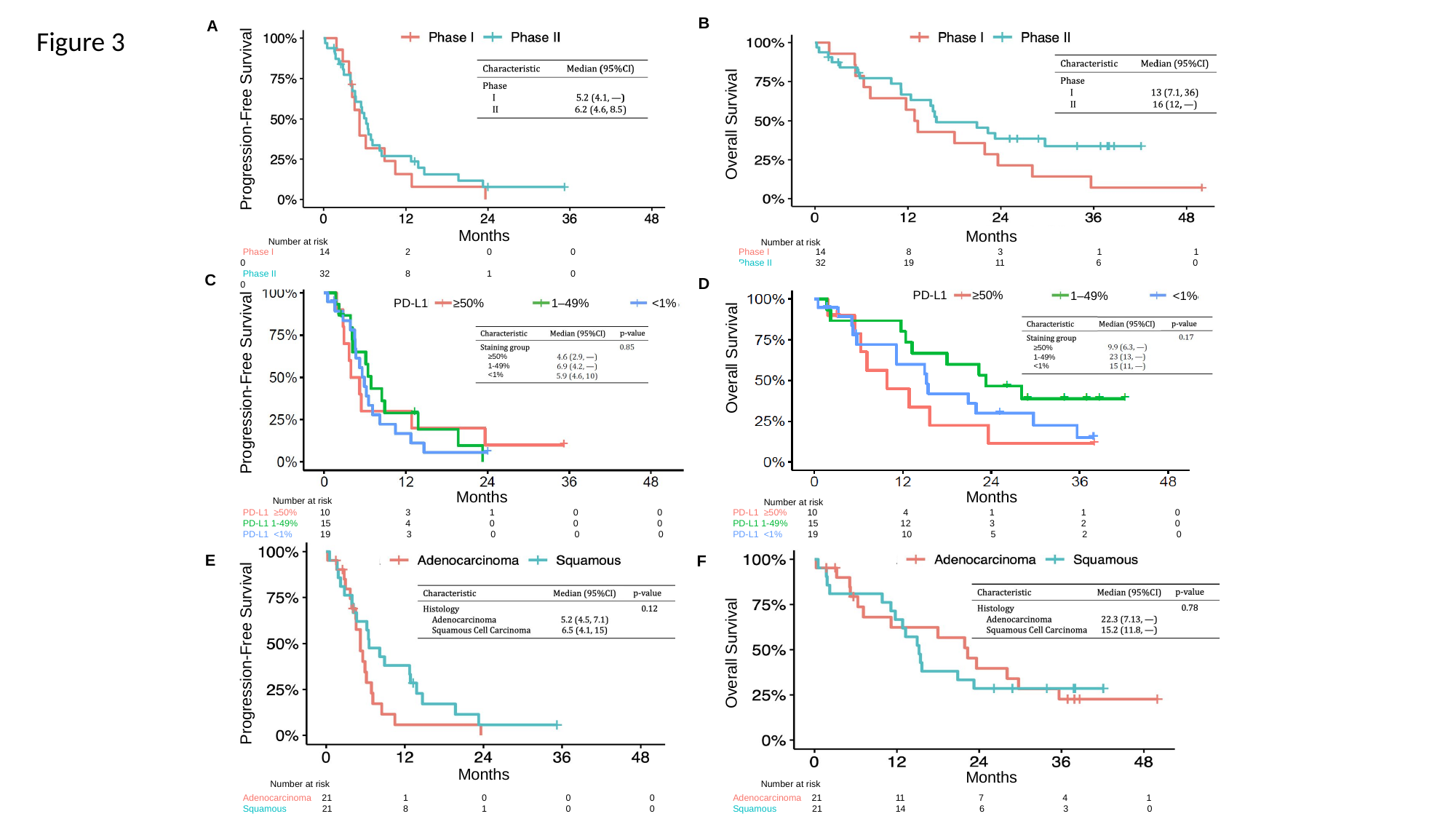

B
A
Figure 3
Progression-Free Survival
Overall Survival
Months
Months
Number at risk
Number at risk
 Phase I 14	 2 0 0 0
 Phase II 32	 8 1 0 0
 Phase I 14	 8 3 1 1
 Phase II 32	 19 11 6 0
Overall Survival
C
D
PD-L1
≥50%
<1%
1–49%
Progression-Free Survival
PD-L1
≥50%
<1%
1–49%
≥50%
1-49%
<1%
≥50%
1-49%
<1%
Months
Months
Number at risk
Number at risk
 PD-L1 ≥50% 10	 3 1 0 0
 PD-L1 1-49% 15	 4 0 0 0
 PD-L1 <1% 19 3 0 0 0
 PD-L1 ≥50% 10	 4 1 1 0
 PD-L1 1-49% 15	 12 3 2 0
 PD-L1 <1% 19 10 5 2 0
E
F
Progression-Free Survival
Overall Survival
Months
Months
Number at risk
Number at risk
 Adenocarcinoma 21	 1 0 0 0
 Squamous 21	 8 1 0 0
 Adenocarcinoma 21	 11 7 4 1
 Squamous 21	 14 6 3 0

## Slide 3
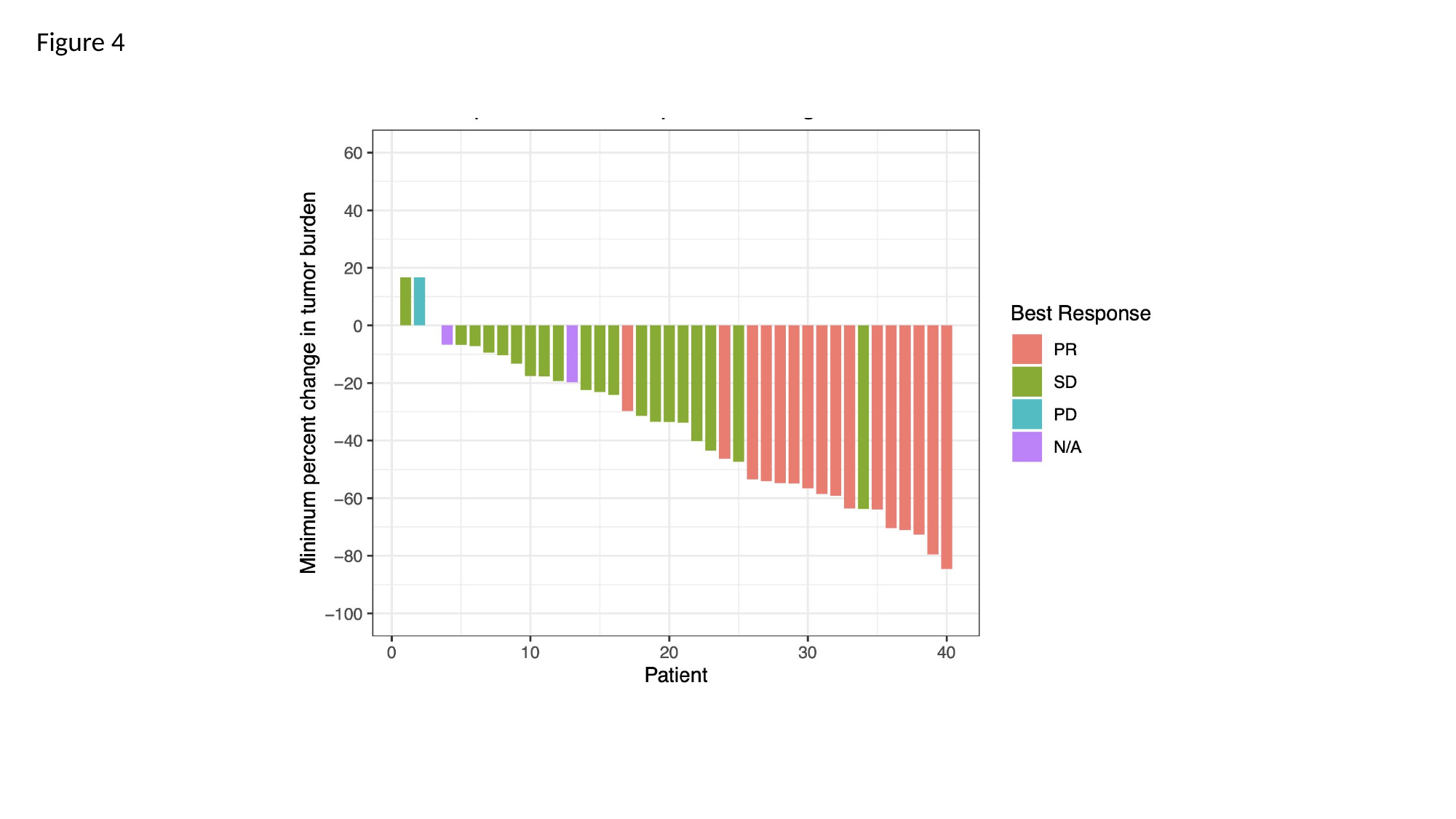

Figure 4

## Slide 4
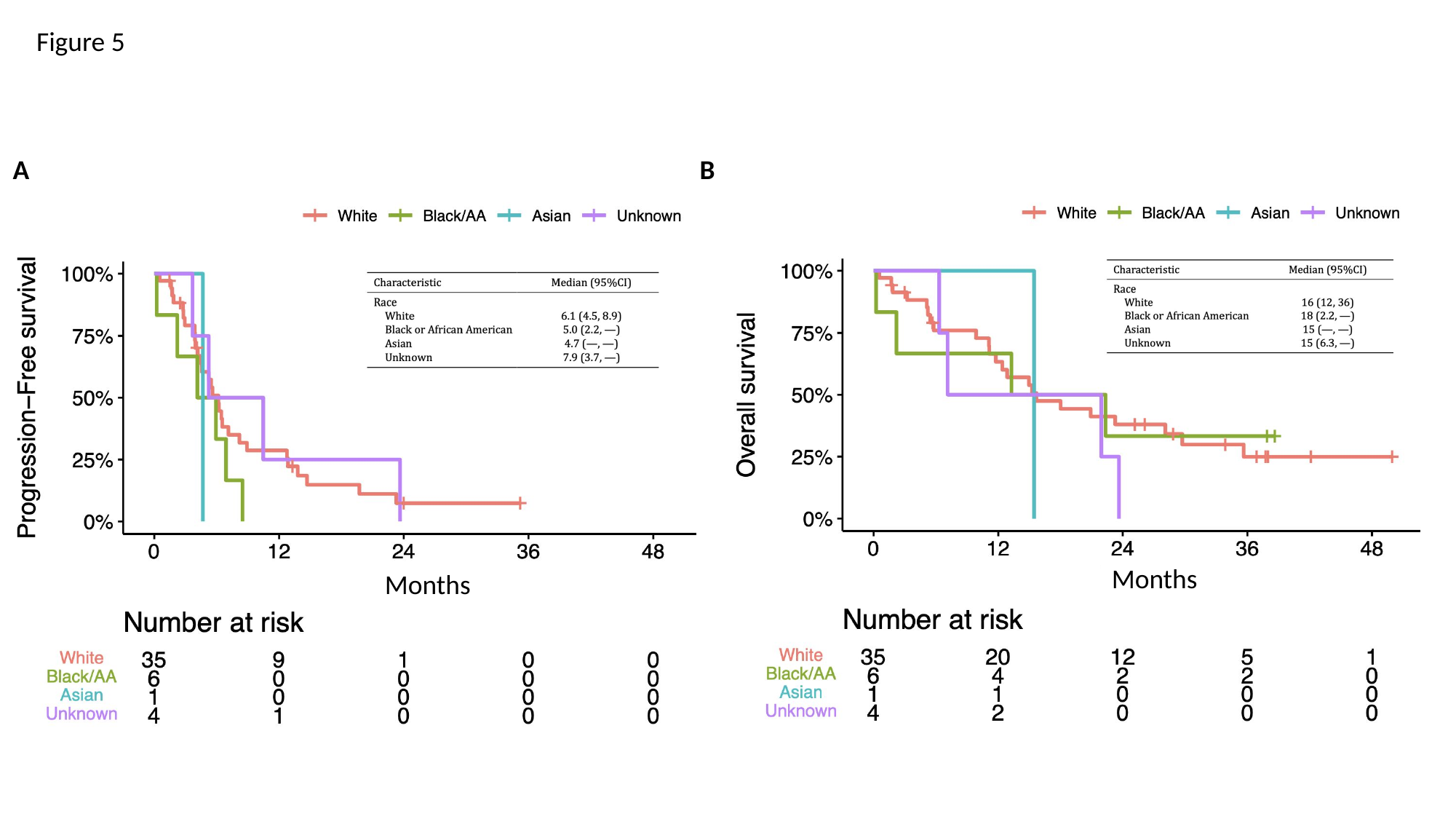

Figure 5
A
B
p=0.45
Months
Months

## Slide 5
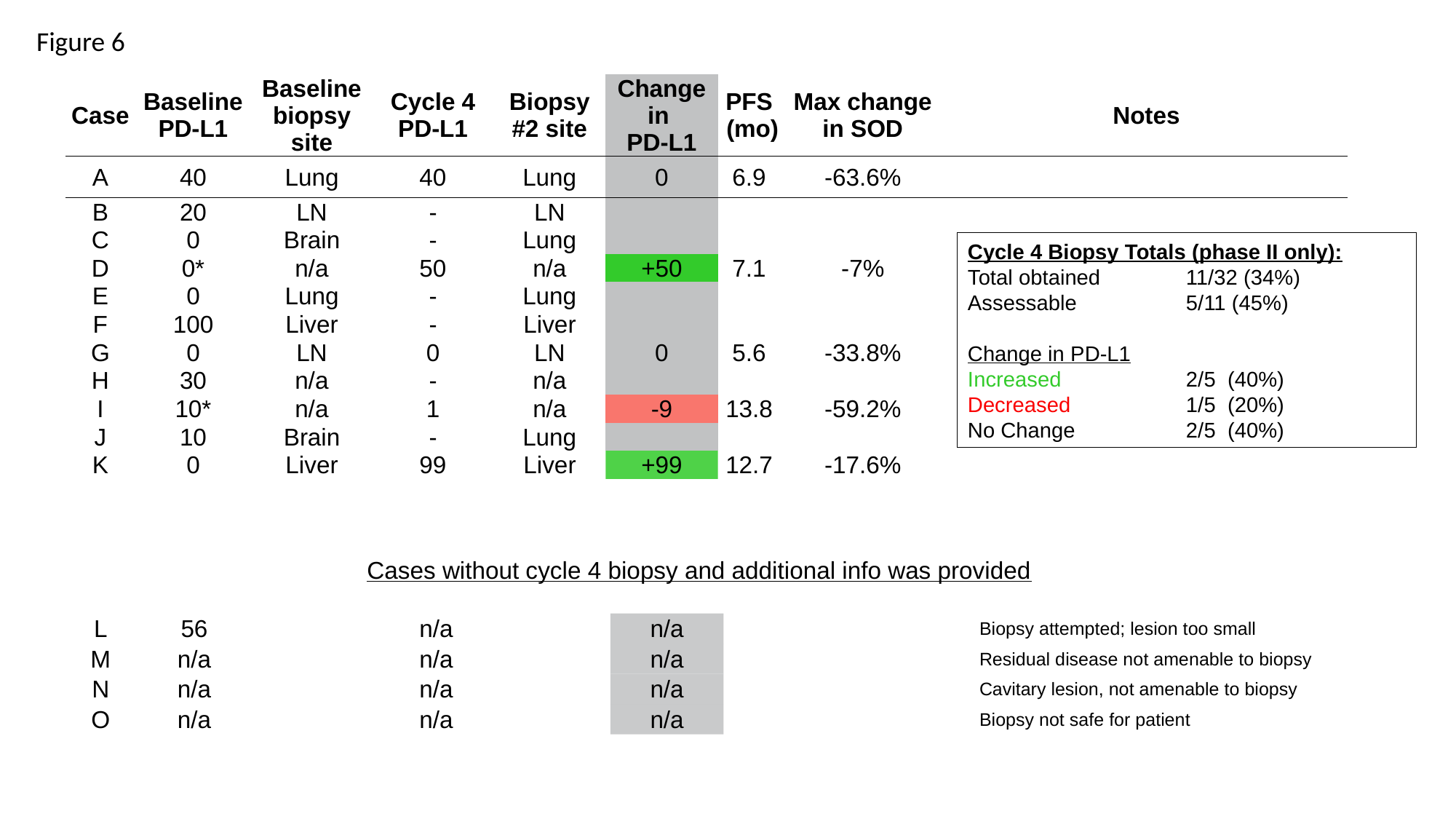

Figure 6
| Case | Baseline PD-L1 | Baseline biopsy site | Cycle 4 PD-L1 | Biopsy #2 site | Change in PD-L1 | PFS (mo) | Max change in SOD | Notes |
| --- | --- | --- | --- | --- | --- | --- | --- | --- |
| A | 40 | Lung | 40 | Lung | 0 | 6.9 | -63.6% | |
| B | 20 | LN | - | LN | | | | |
| C | 0 | Brain | - | Lung | | | | |
| D | 0\* | n/a | 50 | n/a | +50 | 7.1 | -7% | |
| E | 0 | Lung | - | Lung | | | | |
| F | 100 | Liver | - | Liver | | | | |
| G | 0 | LN | 0 | LN | 0 | 5.6 | -33.8% | |
| H | 30 | n/a | - | n/a | | | | |
| I | 10\* | n/a | 1 | n/a | -9 | 13.8 | -59.2% | |
| J | 10 | Brain | - | Lung | | | | |
| K | 0 | Liver | 99 | Liver | +99 | 12.7 | -17.6% | |
Cycle 4 Biopsy Totals (phase II only):
Total obtained	11/32 (34%)
Assessable 	5/11 (45%)
Change in PD-L1
Increased		2/5 (40%)
Decreased 	1/5 (20%)
No Change 	2/5 (40%)
| Cases without cycle 4 biopsy and additional info was provided | | | | | | | | |
| --- | --- | --- | --- | --- | --- | --- | --- | --- |
| L | 56 | | n/a | | n/a | | | Biopsy attempted; lesion too small |
| M | n/a | | n/a | | n/a | | | Residual disease not amenable to biopsy |
| N | n/a | | n/a | | n/a | | | Cavitary lesion, not amenable to biopsy |
| O | n/a | | n/a | | n/a | | | Biopsy not safe for patient |
